# Synergistic effect of Abraxane that combines human IL15 fused with an albumin‐binding domain on murine models of pancreatic ductal adenocarcinoma

**DOI:** 10.1111/jcmm.17220

**Published:** 2022-02-17

**Authors:** Fei‐Ting Hsu, Chang Liang Tsai, I‐Tsang Chiang, Keng‐Hsueh Lan, Po‐Fu Yueh, Wen‐Yi Liang, Chi‐Shuo Lin, Yee Chao, Keng‐Li Lan

**Affiliations:** ^1^ 38019 Department of Biological Science and Technology China Medical University Taichung Taiwan; ^2^ 46615 Department of Biomedical Imaging and Radiological Sciences National Yang Ming Chiao Tung University Taipei Taiwan; ^3^ Medical administrative center Show Chwan Memorial Hospital Changhua Taiwan; ^4^ 63340 Department of Radiation Oncology Show Chwan Memorial Hospital Changhua Taiwan; ^5^ 63340 Department of Radiation Oncology Chang Bing Show Chwan Memorial Hospital Lukang Taiwan; ^6^ Department of Medical Imaging and Radiological Sciences Central Taiwan University of Science and Technology Taichung Taiwan; ^7^ 33561 Department of Oncology National Taiwan University Hospital Taipei Taiwan; ^8^ 33561 Cancer Research Center College of Medicine National Taiwan University Taipei Taiwan; ^9^ 46615 Institute of Traditional Medicine School of Medicine National Yang Ming Chiao Tung University Taipei Taiwan; ^10^ 46615 Department of Pathology Taipei Veterans General Hospital Taipei Taiwan; ^11^ 218818 Department of Radiation Oncology National Yang Ming Chiao Tung University Hospital Yilan Taiwan; ^12^ 46615 Department of Oncology Taipei Veterans General Hospital Taipei Taiwan

**Keywords:** albumin‐binding domain, interleukin‐15, Nab‐paclitaxel (Abraxane), pancreatic adenocarcinoma, tumour microenvironment

## Abstract

Nab‐paclitaxel (Abraxane), which is a nanoparticle form of albumin‐bound paclitaxel, is one of the standard chemotherapies for pancreatic ductal adenocarcinoma (PDAC). This study determined the effect of Abraxane in combination with a fusion protein, hIL15‐ABD, on subcutaneous Panc02 and orthotopic KPC C57BL/6 murine PDAC models. Abraxane combined with hIL15‐ABD best suppressed tumour growth and produced a 40%–60% reduction in the tumour size for Panc02 and KPC, compared to the vehicle group. In the combination group, the active form of interferon‐γ (IFN‐γ)‐secreting CD8^+^ T cells and CD11b^+^CD86^+^ M1 macrophages in tumour infiltrating lymphocytes (TILs) were increased. In the tumour drainage lymph nodes (TDLNs) of the combination group, there was a 18% reduction in CD8^+^IFN‐γ^+^ T cells and a 0.47% reduction in CD4^+^CD25^+^FOXP3^+^ regulatory T cells, as opposed to 5.0% and 5.1% reductions, respectively, for the control group. Superior suppression of CD11b^+^GR‐1^+^ myeloid‐derived suppressor cells (MDSCs) and the induction of M1 macrophages in the spleen and bone marrow of mice were found in the combination group. Abraxane and hIL15‐ABD effectively suppressed NF‐κB‐mediated immune suppressive markers, including indoleamine 2,3‐dioxygenase (IDO), Foxp3 and VEGF. In conclusion, Abraxane combined with hIL15‐ABD stimulates the anticancer activity of effector cells, inhibits immunosuppressive cells within the tumour microenvironment (TME) of PDAC, and produces a greater inhibitory effect than individual monotherapies.

## 1 INTRODUCTION

Pancreatic ductal adenocarcinoma (PDAC) is one of the most significant medical challenges today and has a very poor 5‐year survival rate of <5%.^1^ The poor prognosis for PDAC is mainly due to the difficulty of making an early diagnosis, its resistance to therapies and a high probability of tumour recurrence after treatment.^2^ Since its approval in 1995, gemcitabine monotherapy has been the standard chemotherapy for PDAC patients, but in the following period of more than a decade, numerous clinical trials combining gemcitabine with other chemotherapies failed to demonstrate a significant advantage due to toxicity.^3^, ^4^

Nab‐paclitaxel (Abraxane^®^) is a nanoparticle albumin‐bound form of paclitaxel, with an average size of 130 nm. It was granted FDA approval in 2004 for the treatment of metastatic breast cancer patients for whom combinational chemotherapy is ineffective or who suffer a relapse in less than six months after adjuvant chemotherapy.^5^ A pivotal trial of treatment for metastatic PDAC demonstrated that Abraxane combined with gemcitabine achieved a significant increase in overall survival, progression‐free survival and response rate than gemcitabine monotherapy.^6^

In addition to conventional surgery, chemotherapy and radiotherapy, immunotherapy that uses the immune system to suppress tumour development, and progression is becoming a key element of anticancer therapy. Immune checkpoint inhibitors, including antibodies against CTLA‐4, PD‐1 and PD‐L1, have been successfully used to treat numerous cancer types. However, immunotherapy does not have a significant inhibitory effect on PDAC due to key driver mutations, mediated evasion of immune surveillance, and the fact that immune effector cells cannot efficiently access the tumour microenvironment (TME).^7^, ^8^ The immunosuppressive TME of PDAC is characterized by dense fibrotic tissue, a scarcity of cytotoxic T lymphocytes (CD8^+^ T cells) and natural killer (NK) cells, and abundant regulatory T cells (Treg) and myeloid‐derived suppressive cells (MDSCs).^8^, ^9^ Additionally, the constitutive expression of NF‐κB may form an immunosuppressive TME in various types of cancer.^10^

A lack of infiltration of anticancer effector cells into the TME categorizes PDAC as a ‘cold’ tumour.^11^, ^12^ In this context, it is necessary to determine the effect of immune stimulators combined with proven chemotherapy for the treatment of PDAC. Interleukin‐15 (IL15) is a potent immune stimulator that is critical for the proliferation and activation of natural killer (NK) and CD8^+^ T cells. Wild‐type IL15 and a modified IL15 fusion protein (N‐803), formerly known as ALT‐803 and composed of an N72D IL15 mutant, the sushi domain of IL15Rα, and the human IgG1 Fc domain, have been used in multiple clinical trials with encouraging results.^13^

We previously generated a recombinant protein, hIL15‐ABD, which is human IL15 fused with an albumin‐binding domain (ABD), with increased anticancer efficacy and better results for half‐life, Cmax and area under the curve (AUC) than hIL15. When combined with a rat antibody against a murine PD‐L1 antibody, 10F.9G2, hIL15‐ABD greatly suppressed the growth of subcutaneously inoculated murine CT26 colon rectal carcinoma and B16‐F10 melanoma. This in vivo anticancer effect is associated with an increase in both innate and adaptive anticancer immunity and a decrease in the number of immunosuppressive cells within the TME.^14^

The distinct mechanisms for chemotherapy and immunotherapy mean that a combination of these two presumably complementary strategies may be effective against pancreatic tumours. Herein, Abraxane was combined with hIL15‐ABD to treat two syngeneic pancreatic tumour models: subcutaneous Panc02 and orthotopic KPC in C57/BL mice. This study demonstrates that hIL1‐ABD bound to Abraxane by ABD‐albumin coupling and Abraxane combined with hIL15‐ABD improve the anticancer immune profile within the TME, TDLN, spleen and bone marrow in experimental mice.

## 2 MATERIALS AND METHODS

### 2.1 Cell culture

KPC and Panc02 murine PDAC and CTLL‐2 murine T cell lines were obtained from the Bioresource Collection and Research Center (Hsinchu, Taiwan). KPC and Panc02 cells were respectively cultured in Roswell Park Memorial Institute (RPMI) 1640 medium and Dulbecco's modified Eagle medium (DMEM), supplemented with 10% heat‐inactivated FBS, 2 mM  l‐glutamine, 100 units/ml of penicillin and 100 μg/ml of streptomycin in a humid atmosphere of 5% CO_2_ at 37°C. KPC and Panc02 stably transfected with plasmid encoding both blasticidin‐S deaminase and firefly luciferase (KPC‐luc and Panc02‐luc) were maintained in a medium that was supplemented with 4 ng/ml of blasticidin. CTLL‐2 was cultured in RPMI 1640 supplemented with 10% FBS, 2 mM glutamine, and 100–200 IU/ml of IL2.

### 2.2 Statistical analysis

The results for this study are expressed as the mean ± SEM. Significant differences between multiple groups were determined using a one‐way analysis of variance (ANOVA) and the GraphPad Prism 8.0 software (GraphPad Software, Inc., San Diego, CA). A *p*‐value of <0.05 indicates a significant difference.

The details of the materials and other methods are described in the Supplementary Materials.

## 3 RESULTS

### 3.1 hIL15‐ABD coupled with Abraxane on Panc02 and KPC cells

**FIGURE 1:**
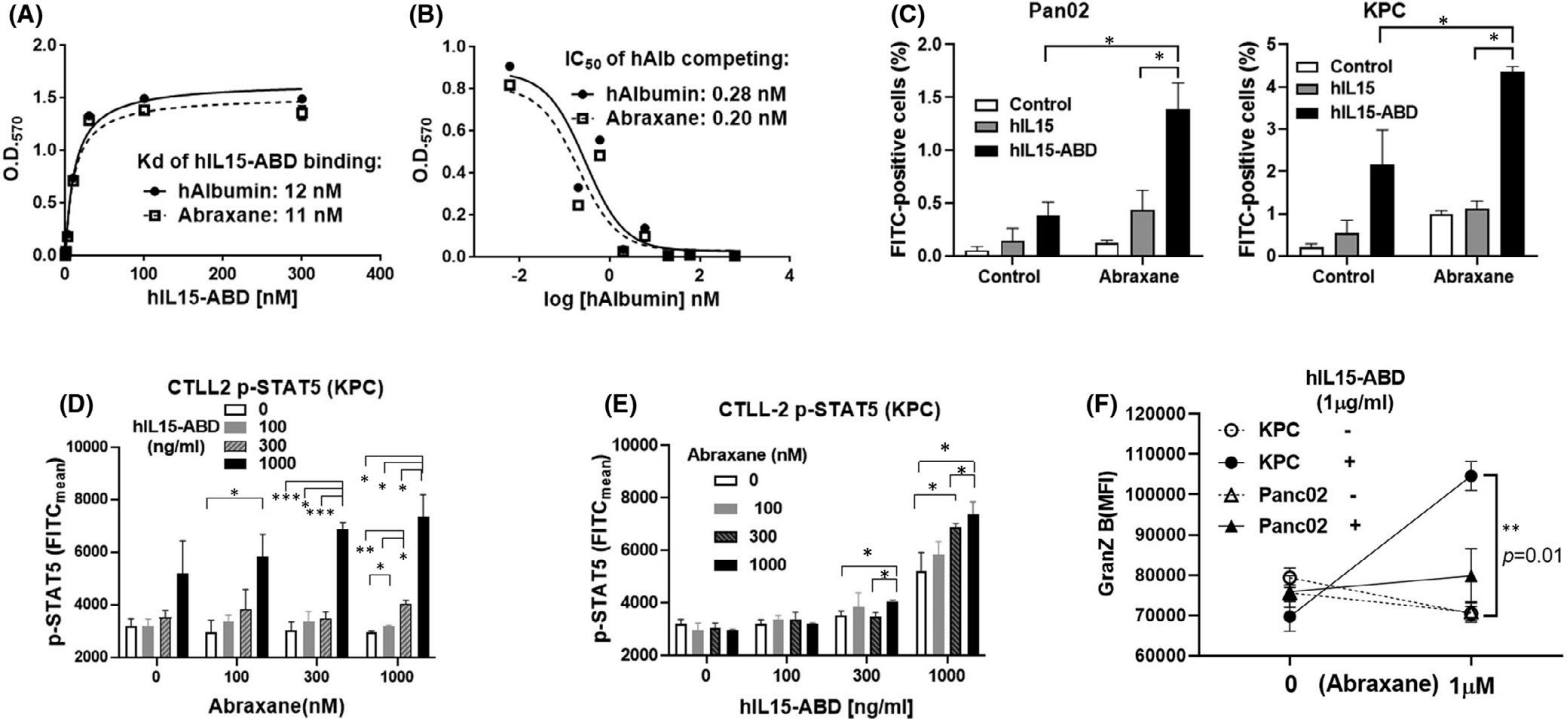
In vitro studies of hIL15‐ABD. (A) ELSA results of increasing concentrations of hIL15‐ABD binding to human albumin (hAlb) and Abraxane that were immobilized on a 96‐well plate. (B) Associations between hIL15‐ABD both hAlb and Abraxane are suppressed as the concentration of competing hAlb increases. (C) Uptake of hIL15 and hIL15‐ABD by either murine PDAC cells was detected by an FITC‐conjugated antibody against his‐tag in the presence or absence of Abraxane using flow cytometry. CTLL‐2 activation was determined by the extent of STAT‐5 phosphorylation after co‐incubation with KPC cells treated with increasing concentrations of (D) Abraxane or (E) hIL15‐ABD. (F) Granzyme B levels in CTLL‐2 cells presented as the mean fluorescence intensity were measured using flow cytometry. The results show decreased cell viability, as determined by luciferase activity (*n* = 3) (*p < 0.05, **p < 0.01 and ***p < 0.001)

In terms of the albumin‐binding capability of ABD, the binding affinity of hIL15‐ABD for Abraxane, which is composed of albumin and paclitaxel at a 9:1 ratio in a nanoparticle form, was determined. The ELISA results displayed similar Kd values of hIL15‐ABD for albumin and Abraxane at 12 and 11 nM respectively (Figure 1A). In the presence of competing human albumin, hIL15‐ABD binding to albumin or Abraxane immobilized on the ELISA plate was suppressed in a dose‐dependent manner (Figure 1B). The uptake of hIL15 and hIL15‐ABD by murine PDAC cells either in the presence or absence of Abraxane was detected by flow cytometry. As opposed to hIL15‐ABD, hIL15 did not show significant Abraxane‐enhanced uptake by Pan02 and KPC (Figure 1C).

To determine whether hIL15‐ABD coupled to Abraxane stimulates immunity, CTLL‐2 cells were incubated with KPC in the presence or absence of these two therapeutics. The greatest degree of STAT‐5 phosphorylation, which represents immune activation, was achieved if CTLL‐2 cells were co‐cultured with KPC pre‐treated with a concentration of Abraxane in the range of 300–1000 nM (Figure 1D) and hIL15‐ABD in the range of 300–1000 ng/ml (Figure 1E). Activation of CTLL‐2 cells was also examined by detecting any increase in Granzyme B. Only in the presence of KPC cells did Abraxane combined with hIL15‐ABD result in a moderate increase from the high baseline level of Granzyme B in CTLL‐2 cells (Figure 1F).

### 3.2 hIL15‐ABD enhanced the anti‐tumour efficacy of Abraxane in xenograft Panc02 murine PDAC models

**FIGURE 2:**
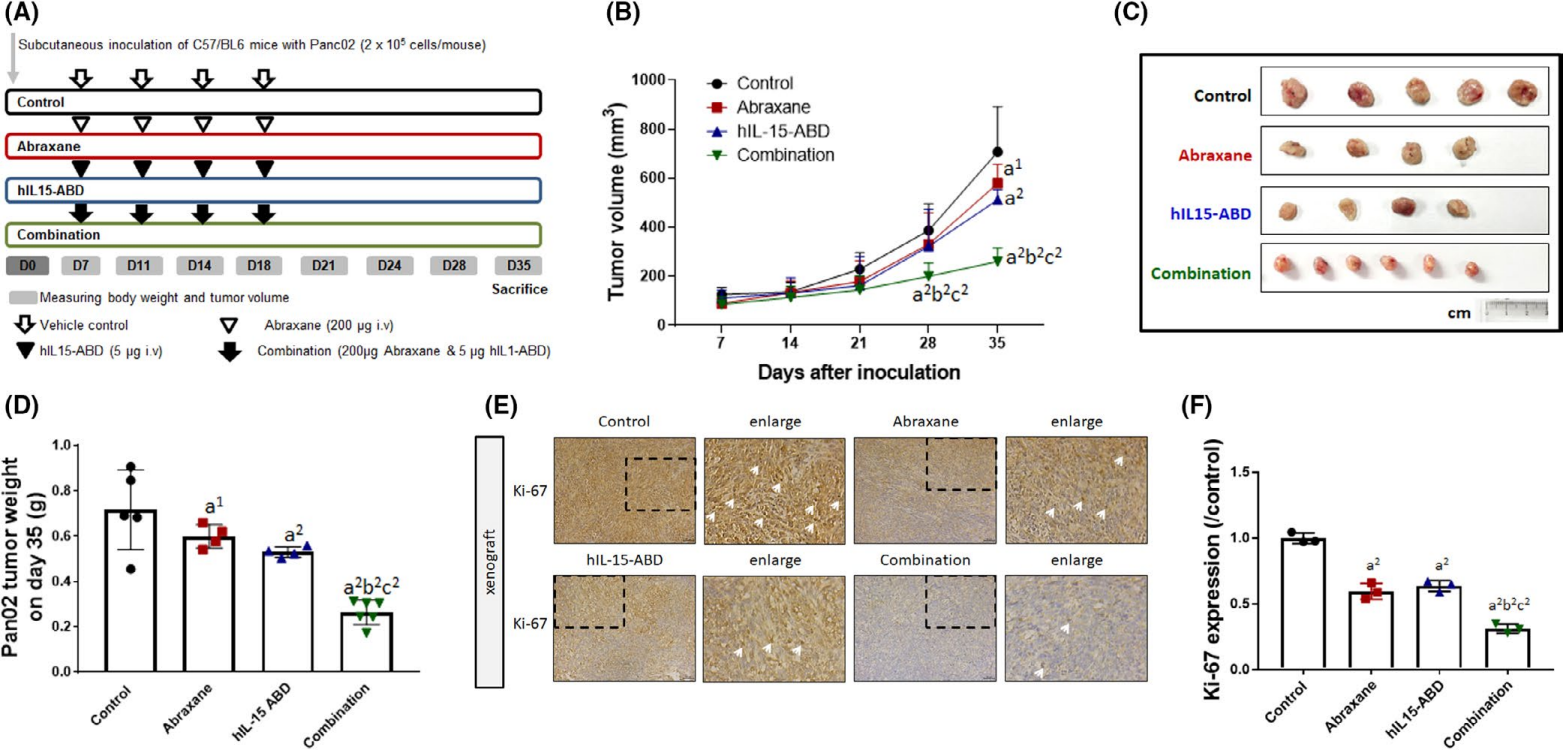
Anti‐tumour efficacy of Abraxane was enhanced by combination with hIL15‐ABD in subcutaneous Panc02 models. (A) An experimental flow chart of the Panc02 xenograft animal model. (B) Tumour growth, (C) extracted tumour image and (D) extracted tumour weight are displayed. (E) IHC of Panc02 expressing Ki‐67 and (F) quantification data of Ki‐67 are shown (a^1^ and a^2^ vs. control *p* < 0.05 and *p* < 0.01; b^2^ vs. Abraxane, *p* < 0.01; c^2^ vs. hIL15‐ABD, *p* < 0.01)

Panc02 cells bearing the xenograft model were used to examine the treatment efficacy of hIL15‐ABD and Abraxane. Mice were treated with the vehicle control, hIL15‐ABD (5 μg), Abraxane (200 μg) or a combination twice per week for two weeks by intravenous injection (Figure 2A). The tumour volumes were measured until one week after the last injection. Figure 2B shows that the tumours did not progress as quickly for the combination group as for each single treatment. Abraxane combined with hIL15‐ABD led to the best results for the mean tumour‐doubling time, delay time, inhibition rate and enhancement ratio (Table 1). A combination index of 0.88 was calculated using the Chou‐Talalay method,^15^ so Abraxane combined with hIL15‐ABD synergistically inhibited tumour growth in the subcutaneous Panc02 group (Table 2). Figure 2C shows that the combination exhibited enhanced tumour growth inhibition. This was also demonstrated in tumours that were excised from mice on Day 35 after Panc02 inoculation. The average weight of an excised tumour for the combination group was statistically lower than for the single treatment groups (Figure 2D). To determine the anti‐tumour effect of the combination therapy, the expression level of the proliferation marker Ki‐67 was determined using IHC (Figure 2E). Ki‐67 was expressed less in the xenograft Panc02 for mice that were treated with Abraxane combined with hIL15‐ABD than those that were treated using the monotherapies (Figure 2F). This shows that hIL15‐ABD sensitizes Panc02 to Abraxane.

**TABLE 1:** Mean tumour growth time, delay time, inhibition rate and enhancement ratio in xenograft Panc02 tumour‐bearing mice after treatment with Abraxane, hIL‐15‐ABD and the combination of both

Treatment	MTGT (day)^a^	MTGDT (day)^b^	MGIR^c^	ER^d^
Control	12.03	NA	NA	NA
Abraxane	14.30	2.27	1.19	2.80
hIL15‐ABD	17.49	5.46	1.45	2.29
Combination	40.11	28.09	3.33	

Abbreviation: NA, not available

^a^ Mean tumour growth time (MTGT): the time at which the tumour volume reached 500 mm^3^.

^b^ Mean tumour growth delay time (MTGDT): the mean tumour growth time of the treated group minus that of the control group.

^c^ Mean growth inhibition rate (MGIR): the mean tumour growth time of the treated group/the mean tumour growth time of the sham control group.

^d^ Enhancement ratio (ER): the mean growth inhibition rate of combination group/the mean growth inhibition rate of Abraxane or hIL15‐ABD group.

**TABLE 2:** Mean tumour growth inhibition rate and combination index in Panc02 tumour‐bearing mice after treatment with Abraxane, hIL15‐ABD and the combination of both

Treatment	Mean inhibitory^a^	Expected inhibitory^b^	Combination index^c^
Control	–	–	–
Abraxane	0.18	–	–
hIL‐15‐ABD	0.28	–	–
Combination	0.63	0.58	0.88 (synergistic effect)

^a^ Mean growth inhibitory rate: 1−(the 14th day's mean tumour volume ratio of treated group/the 14th day's mean tumour volume ratio of the sham control group).

^b^ Expected growth inhibitory rate: inhibition rates of combination minus the multiplication of both Abraxane, IL‐15‐ABD inhibition rates.

^c^ Combination index: (1−Mean growth inhibitory rate of combination)/(1−Expected growth inhibitory rate).

### 3.3 hIL15‐ABD enhanced the anti‐tumour efficacy of Abraxane in orthotopic KPC murine PDAC models

**FIGURE 3:**
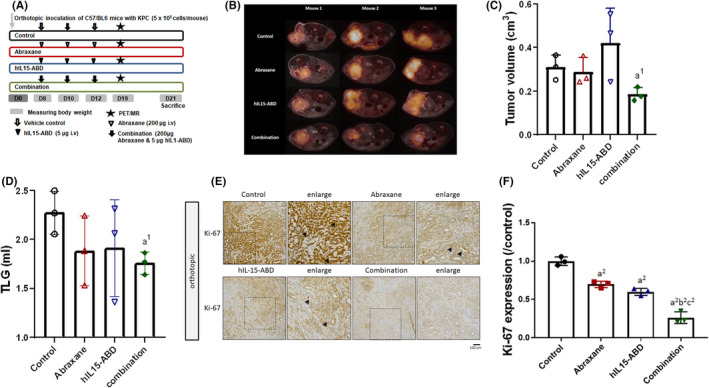
Growth of orthotopic KPC PDAC was significantly suppressed by Abraxane combined with hIL15‐ABD. (A) An experimental scheme of the orthotopic KPC model. (B) Representative PET/MRI images for orthotopic KPC PDAC seven days after the last of the three intravenous injections of the vehicle control, Abraxane, hIL1‐ABD and the combination. (C) Tumour volumes as determined based on the PET/MRI images. (D) Total lesion glycolysis (TLG) was calculated by the tumour volume times the SUV mean. (E) IHC of the Ki‐67 expression in orthotopic KPC tumours and (F) quantification data of Ki‐67 are shown (a^1^ and a^2^ vs. control, *p* < 0.05 and *p* < 0.01; b^2^ vs. Abraxane, *p* < 0.01; c^2^ vs. hIL15‐ABD, *p* < 0.01)

The anticancer efficacy of Abraxane and hIL15‐ABD was determined for the KPC pancreatic orthotopic model using PET/MRI (Figure 3A). The tumour volumes were determined by contouring the gross tumour. The MRI‐ and PET‐segmented images showed that tumour growth in the mice treated with a combination of Abraxane and hIL15‐ABD was lower than that for the control group or the group treated with a monotherapy of Abraxane or hIL15‐ABD (Figure 3B,C). The images for the Abraxane hIL15‐ABD combination group [^18^F]‐FDG also displayed the greatest reduction in glucose uptake, which is shown as total lesion glycolysis (TLG), as determined by the tumour volume times the SUV mean (Figure 3D). The expression patterns for the Ki‐67 marker in orthotopic KPC were the same as those for subcutaneous Panc02‐bearing mice for the different treatment groups, as shown in Figure 3E. Abraxane combined with hIL15‐ABD better suppressed Ki‐67 expression than an individual monotherapy using either Abraxane or hIL15‐ABD (Figure 3F).

### 3.4 Abraxane combined with hIL15‐ABD potentiated anticancer immunity in murine PDAC models

**FIGURE 4:**
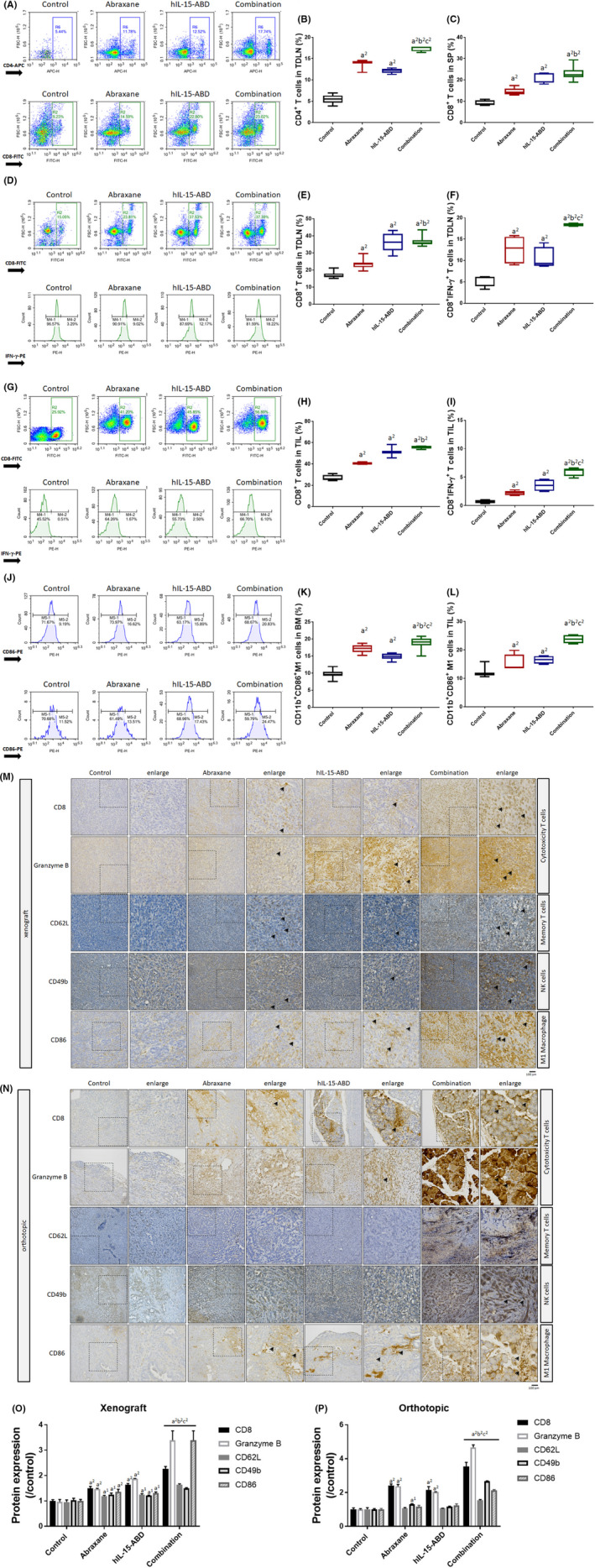
Population and function of cytotoxic T cells and M1 macrophage were increased by a combination of Abraxane and hIL15‐ABD. (A) The expression pattern and quantification results of (B) CD4^+^ T cells in TDLN and (C) CD8^+^ T cells in SP are shown. (D) The expression pattern and quantification results of (E) CD8^+^ T cells and (F) CD8^+^ IFN‐γ^+^ T cells in TDLN are investigated. (G) The expression pattern and quantification results of (H) CD8^+^ T cells and (I) CD8^+^ IFN‐γ^+^ T cells from TIL are displayed. (J) The expression pattern and quantification results of (H) CD11b^+^CD86^+^ M1‐like macrophage in BM and (I) CD11b^+^CD86^+^ M1‐like macrophage in TIL are displayed. (M,N) The xenograft and orthotopic IHC‐stained images and (O,P) quantification results of CD8, Granzyme B, CD62L, CD49b and CD86 are displayed (a^2^ vs. control, *p* < 0.01; b^2^ vs. Abraxane, *p* < 0.01; c^2^ vs. hIL15‐ABD, *p* < 0.01)

To determine the local and systemic immune activation resulting from hIL15‐ABD and Abraxane, an analysis of the immune population within the spleen,^16^ bone marrow (BM), tumour‐draining lymph nodes (TDLNs) and tumour infiltrating lymphocytes (TILs) of the mice was conducted. Figure 4A,B shows that CD4^+^ T cells, which represented the memory population of T cells, increased significantly for the group treated with Abraxane combined with hIL15‐ABD. The cytotoxic T‐cell population CD8^+^ T cells was also increased for the combination group (Figure 4A,C). The population and activation of cytotoxic T cells in TDLNs was increased by the combination treatment (Figure 4D,E). IFN‐γ, which is an intracellular marker of CD8^+^ T cell activation, was significantly increased in the combination group, compared to each single treatment (Figure 4D,F). There was systemic immune potentiation in the combination group and an increase in the population and activation of cytotoxic T cells in the tumour microenvironment. The population of CD8^+^ T cells increased from 25.9% to 56.8% after combination therapy (Figure 4G,H). The activation marker IFN‐γ of the CD8^+^ T cells in the TILs increased 12 times more in the combination group than in the control group (Figure 4G,I). The common resource of macrophages is bone marrow; thus, the type of macrophages that expanded after treatment was validated by flow cytometry. The populations of CD11b^+^CD86^+^ M1‐like macrophages in BM and TILs also increased in the combination therapy group (Figure 4J–L).

We also performed IHC staining on both Pan02 subcutaneous and KPC orthotopic tumour tissues to examine the expression level of various markers of immune activation, including CD8, granzyme B, CD62L, CD49b and CD86. The protein expression of CD8 and Granzyme, representing cytotoxicity T cell accumulation and function, respectively, increased in the combination group (Figure 4M–P). The memory T and NK markers, including CD62L and CD49b, also increased in the combination group (Figure 4M–P). As shown in Figure 4M‐P, the CD86 expression was significantly induced by the hIL15‐ABD and Abraxane combination, consistent with the flow cytometry data displaying an accumulation of CD11b^+^CD86^+^ M1‐like macrophages within tumours from mice of the combination group. These flow cytometry and IHC results suggest that Abraxane marginally increases the accumulation of CD8 M1‐like macrophages, as well as cytotoxicity in the immune system, both systemically (spleen, bone marrow and TDLNs) and locally (TME). Moreover, the combination with hIL15‐ABD further enhanced the anticancer immunity of Abraxane, evidenced by the increased accumulation and activity of anticancer immune cells.

### 3.5 Abraxane combined with hIL15‐ABD attenuated the NF‐κB‐mediated immunosuppressive effect on murine PDAC models

**FIGURE 5:**
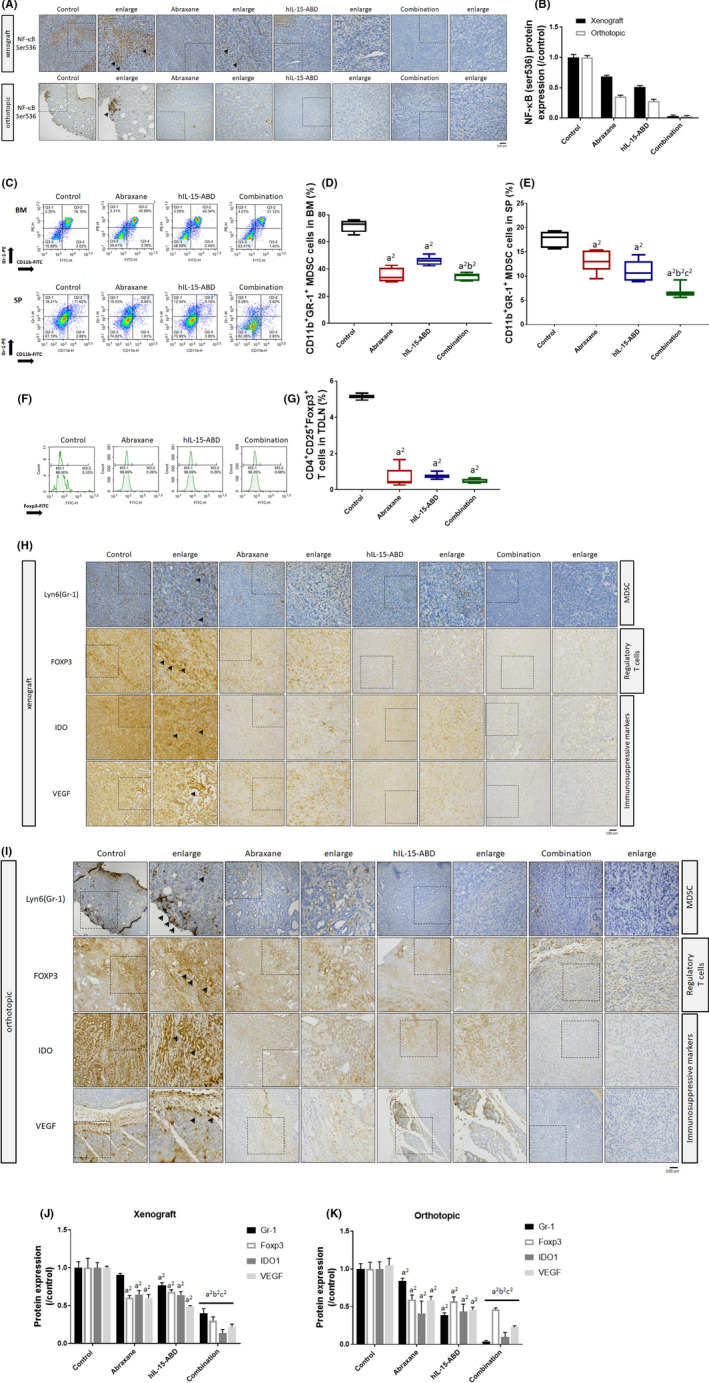
Population and function of MDSCs and Treg cells were inhibited by Abraxane combined with hIL15‐ABD. (A,B) The protein expression and quantification results of NF‐κB (ser536). (C) The expression pattern and quantification results of MDSCs in (D) BM and in (E) SP are shown. (F) The expression pattern and (G) quantification results of CD4^+^CD25^+^Foxp3^+^ Tregs were determined in TDLN. (H,I) IHC‐stained images and (J,K) quantification results of Gr‐1, Foxp3, IDO and VEGF are displayed (a^1^ and a^2^ vs. control, *p* < 0.05 and *p* < 0.01; b^1^ and b^2^ vs. Abraxane, *p* < 0.05 and *p* < 0.01; c^1^ and c^2^ vs. hIL15‐ABD, *p* < 0.05 and *p* < 0.01)

First, we proved that the phosphorylation of NF‐κB, an important transcription factor that is involved in the immunosuppressive effect in the TME, was decreased after the combination treatment (Figure 5A,B). Then, to examine the effect of Abraxane combined with hIL15‐ABD on immunosuppressive cells, the extent of myeloid‐derived suppressor cells (MDSCs) presenting in the bone marrow (BM) and spleen^16^ of Panc02 tumour‐bearing mice treated with monotherapies of Abraxane and hIL15‐ABD, or a combination thereof, were evaluated using flow cytometry. Figure 5C–E shows that the MDSCs in the bone marrow and spleen were reduced by treatment with Abraxane combined with hIL15‐ABD, compared to the control group. Foxp3, which is a marker od activated Treg cells, was suppressed in the TDLNs upon combination therapy (Figure 5F,G). Similar IHC results for Foxp3 were also seen for the xenograft and orthotopic tumour tissues (Figure 5H–K). The expression levels of other immune suppressive factors, such as IDO and VEGF, significantly decreased following a combination treatment of Abraxane and hIL15‐ABD (Figure 5H–K). These results show that Abraxane combined with hIL15‐ABD inhibits immunosuppression.

### 3.6 hIL15‐ABD enhanced Abraxane‐induced extrinsic and intrinsic apoptosis signalling in both Panc02 and KPC PDAC models

**FIGURE 6:**
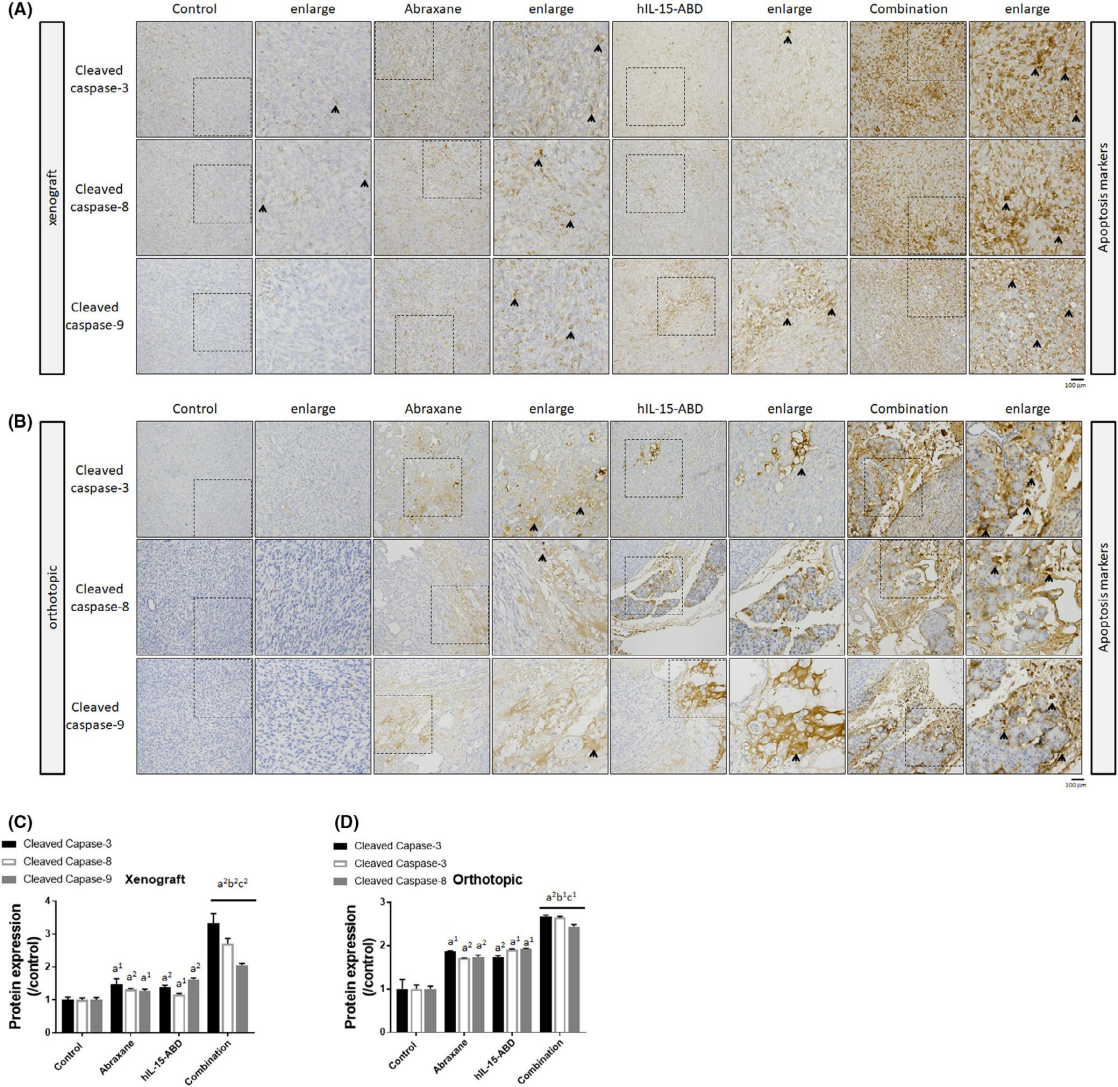
Extrinsic and intrinsic apoptotic pathway induced by Abraxane was promoted by hIL15‐ABD. (A,B) IHC images and quantification data of, and (C,D) quantification results for, cleaved caspase‐3, −8 and −9 (a^2^ vs. control, *p* < 0.01; b^2^ vs. Abraxane, *p* < 0.01; c^2^ vs. hIL15‐ABD, *p* < 0.01)

To determine whether apoptosis signalling is activated by treatment, the protein expression of cleaved caspase‐3, ‐8 and ‐9 in both xenograft and orthotopic tumour tissues was calculated. Figure 6A,B shows that the protein expression level of cleaved caspase‐3 was 1.6 times greater in the combination group than in the control group. There was also a 1.5 times more of the extrinsic apoptosis marker cleaved caspase‐8 in the combination group (Figure 6C,D). The protein expression of cleaved caspase‐9, involved in the intrinsic apoptosis pathway, was triggered by Abraxane combined with hIL15‐ABD. The level of expressed apoptotic protein was greater for the combination treatment for the xenograft and orthotopic animal models.

### 3.7 An effective dose of Abraxane combined with hIL15‐ABD did not induce a pathological change in the normal organs of PDAC‐bearing mice

**FIGURE 7:**
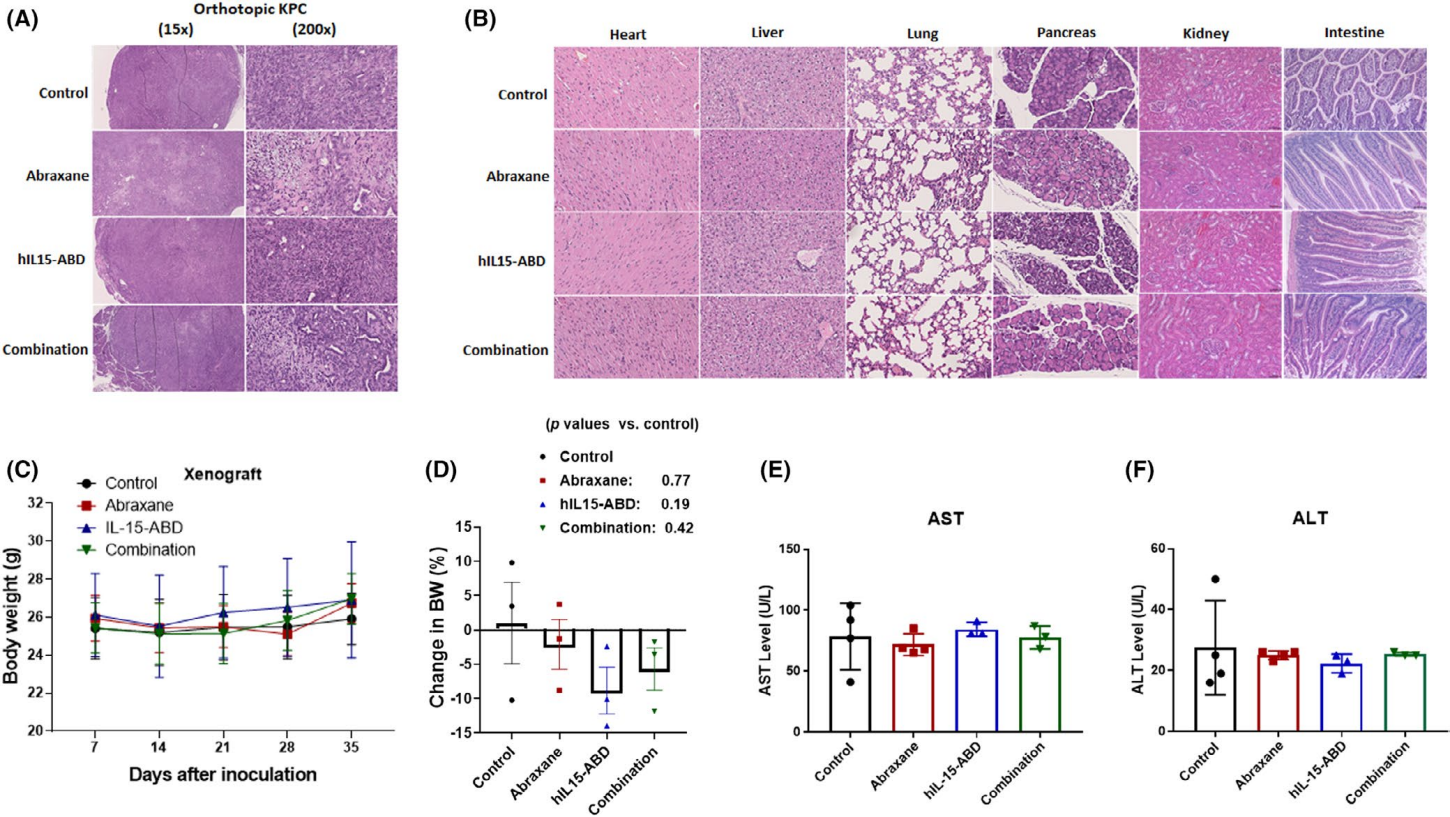
No discernible general or normal organ toxicity was identified in mice treated with both Abraxane and hIL15‐ABD. (A) Representative haematoxylin and eosin staining image (15× and 200× magnifications) of an orthotopic KPC tumour and (B) representive haematoxylin and eosin‐stained normal organs (200× magnification). Body weight (BW) of Panc02 xenograft mice from Days 7 to 35: (D) BW of the mice inoculated with KPC on Day 0 was used as the baseline, and the difference in BW was determined on Day 19, when the KPC‐bearing mice were subjected to PET/MR imaging. The serum level of (E) AST and (F) ALT of the Panc02 xenograft model

The images for HE‐stained orthotopic KPC are different for each treatment group. Mice treated with either Abraxane only or Abraxane combined with hIL15‐ABD had more extensive fibrosis and necrotic regions, but those treated with either hIL5‐ABD only or a combination of Abraxane and hIL15‐ABD exhibited significant infiltration of inflammatory cells. However, the orthotopic KPC tumours of mice treated with the vehicle exhibited limited necrosis and fibrosis, and there was little infiltration of the inflammatory cells (Figure 7A). hIL15‐ABD induced more significant infiltration of immune cells in orthotopic KPC tumours, but there was no evidence of a similar phenomenon within the normal organs, such as the heart, lungs, liver, kidneys, intestines and pancreas, adjacent to the KPC tumour (Figure 7B). The body weight of subcutaneous Panc02‐ (Figure 7C) and orthotopic KPC‐bearing mice (Figure 7D) showed no statistically significant difference between groups. The enzyme levels of AST, ALT (Figure 7E,F) and γGT (Table 3) were not significantly different for the mice from each treatment group, so the general condition and liver function of the experimental mice was not affected by Abraxane, hIL15‐ABD or a combination thereof.

**TABLE 3:** The serum level of gamma‐glutamyl transferase (γGT)

Group	γGT (U/L)
Ctrl‐1	<3
Ctrl‐2	<3
Ctrl‐3	<3
Abraxane‐1	<3
Abraxane‐2	<3
Abraxane‐3	<3
IL‐15‐ABD‐1	<3
IL‐15‐ABD‐2	<3
IL‐15‐ABD‐3	<3
Combination‐1	<3
Combination‐2	<3
Combination‐3	<3

## 4 DISCUSSION

Gemcitabine is a standard chemotherapy for the majority of PDAC patients and was approved in 1995. Gemcitabine was the preferred treatment for PDCA until 2011, when the FOLFIRINOX regimen (oxaliplatin, irinotecan, leucovorin and 5‐fluorouracil) resulted in better overall survival in a randomized clinical trial for patients with metastatic pancreatic cancer.^17^ However, the serious adverse effects mean that FOLFIRINOX is only suitable for PDAC patients with a relatively good performance status. Abraxane is a unique nanoparticle of an albumin‐bound form of paclitaxel, which has a higher response rate and enhanced safety profiles compared to a solvent‐based formulation of paclitaxel.^18^, ^19^ Abraxane is associated with a prolonged half‐life, increased accumulation in the tumour, macropinocytosis‐driven activation of macrophages, apoptosis of cancer cells and improved accessibility for immune cells and therapeutics to the TME through stromal depletion.^20^ The treatment of Abraxane combined with gemcitabine was granted FDA approval in 2013 for patients with late‐stage pancreatic cancer, based on the results of a phase III randomized trial, which demonstrated that Abraxane/gemcitabine provides a significantly improved median overall survival of 8.5 months, compared to 6.7 months for gemcitabine alone.^5^ However, the prognosis for PDAC patients is very poor, and this remains a significant challenge.

Although immunotherapy has revolutionized the treatment of numerous types of cancer, there is no definitively positive result for PDAC patients.^21^ PDAC is relatively resistant to chemotherapy or immunotherapy, mainly due to its characteristic TME with a fibrotic barrier formed by collagen, fibronectin, and hyaluronic acid, which prevents the delivery of sufficient chemodrugs and infiltration of immune effector cells.^22^ The lack of NK and CD8^+^ T cells within the TME illustrates PDAC as a ‘cold’ tumour.^11^, ^12^ Therefore it is plausible to turn a toxic drug‐challenged PDAC into a ‘hot’ tumour with the addition of immune stimulants, which enhance the infiltration of active immunity in the TME.

In addition to tumour cells, which are the major target of conventional anticancer therapy, stromal, endothelial, and immune cells all play critical roles in the progression, survival and metastasis of tumour cells. Nanomedicine is an increasingly common strategy for targeting the TME with multiple complementary therapeutics,^23^, ^24^, ^25^ including cytotoxic drugs, radiation, and immunotherapy. Liposomal doxorubicin (Doxil) and Abraxane, which have an average respective size of around 100 and 125 nm, are some of the first nanoparticle chemotherapies in clinical use. As opposed to normal organs with tight interendothelial junctions, tumours are often associated with disorganized blood vessels with gaps between endothelial cells of up to a few micrometres.^26^ Therefore, the sizes of Doxil and Abraxane allow improved tumour localization and a better safety profile due to an enhanced permeability retention (EPR) effect.^27^, ^28^

Some chemodrugs, such as doxorubicin and paclitaxel, which are major components of Doxil and Abraxane, respectively, can trigger immunogenic cell death (ICD) within the TME,^29^ but chemodrug‐induced anticancer immunity has not been frequently demonstrated for long‐term tumour suppression. It is becoming evident that the cancer–immunity cycle involves multiple key steps,^30^ and a combination of complementary therapeutics may be required to advance different immune regulatory steps, including tumour‐killing, antigen release, processing and presentation, activation and infiltration of immune effect cells and inhibition of immune checkpoints. Although Abraxane was shown to modulate anticancer immunity through inducing ICD, immune modulators capable of regulating complementary immune key steps could further promote the cancer–immunity cycle. Several preclinical studies have shown encouraging results in studies combining solvent‐based paclitaxel or Abraxane with immune modulators, such as IL2,^31^, ^32^ F8‐IL2,^16^, ^33^ IL12,^34^, ^35^, ^36^ PD‐L1 ^37^, ^38^ and PD‐1^39^, ^40^ antibodies. Moreover, a recent phase III clinical study used Abraxane combined with the PD‐L1 antibody atezolizumab to treat patients with metastatic triple‐negative breast cancer, and demonstrated prolonged progression‐free survival for these patients in the absence of more significant adverse events, compared to the results for each monotherapy.^41^ Abraxane combined with PD‐1 antibodies, including pembrolizumab, nivolumab, toripalimab and camrelizumab, was also shown to be a well‐tolerated and effective regimen for 64 Chinese patients with refractory melanoma.^42^ A retrospective study showed a significantly improved median overall survival of 28.6 months for 17 patients who received both Abraxane and the PD‐1/PD‐L1 inhibitor. After platinum‐based chemotherapy, the median overall survival was 15.9 months for 40 patients who were treated with the PD‐1/PD‐L1 inhibitor only for progressed non‐small cell lung cancer (NSCLC).^43^

To further potentiate the anticancer immunity of Abraxane on PDAC by modulating anticancer immunity, studies have used Abraxane in the presence or absence of gemcitabine in combination with therapeutics in multiple clinical settings. In a randomized phase II study involving 34 pre‐operative pancreatic cancer patients, high‐dose hydroxychloroquine combined with gemcitabine/nab‐paclitaxel was shown to increase the TME immune infiltration score, which is associated with significantly improved (*p* < 0.0001) progression‐free and overall survival, compared to the results of 30 patients who received only gemcitabine/nab‐paclitaxel. Signs of enhanced anti‐PDAC efficacy for gemcitabine/nab‐paclitaxel using the PD‐1 antibodies toripalimab and pembrolizumab were evident in patients with a durable response^44^ and a 100% disease control rate (27% PR + 73% SD) for 11 evaluable patients.^45^

In light of the potential efficacy of Abraxane combined with immunotherapy, tens of clinical trials of this combinational regimen for patients with PDAC have been undertaken, as shown in the search results of the www.clinicaltrials.gov database using a search with keywords including pancreatic cancer, nab‐paclitaxel and immunotherapy. The immunotherapies combined with Abraxane in PDAC clinical trials include PD‐1 antibodies (pembrolizumab, nivolumab, toripalimab^46^ and cemiplimab), PD‐L1 antibodies (durvalumab, avelumab and atezolizumab), CTLA‐4 antibody (ipilimumab), CD40 agonist (selicrelumab and sotigalimab^47^), CXCR4 inhibitor (BL‐8040), CEA vaccine (ETBX‐011), mutant RAS vaccine (GI‐4000), indoleamine 2,3‐dioxygenase‐1 inhibitor (indoximod), NK cell line (haNK) and IL15 superagonist (N‐803).

Interleukin‐2 plays a positive role in the development and expansion of T cells and was approved for the treatment of metastatic melanoma and renal cell carcinoma more than two decades ago.^48^ However, there is increasing evidence of the limitations of IL2 as an anticancer immunotherapeutic. IL2 binds with high affinity to IL2Rα, which is abundantly expressed by regulatory T cells (Treg), and this stimulates immunosuppression at a lower dose. To enhance anticancer immunity for IL2Rβγ‐expressing NK and CD8 cells, the risk of pulmonary oedema is associated with a higher IL2 dose.^48^ IL2 also contributes to Fas‐mediated activation‐induced cell death (AICD) of T cells, which is critical for peripheral self‐tolerance. These immunosuppressive characteristics of IL2 mean that it has a relatively narrow therapeutic window as an anticancer therapeutic.

IL15 is one of the type I family members of IL2. It supports the proliferation, activation and survival of natural killer (NK) and CD8^+^ T cells in the absence of Treg activation and plays no role in AICD.^49^ Given its positive effects on anticancer immunity, IL15 was ranked as the most promising immunotherapy for cancer treatment by an immunologist panel at a National Cancer Institute workshop.^50^ In its first dose‐escalating clinical trial, IL15 was intravenously administered for 12 consecutive days to patients with metastatic renal cell carcinoma and melanoma, and its efficacy was evident in some patients with the clearance of metastatic lesions.^51^ Fc fused with a complex of IL15 and the IL1Rα sushi domain (IL15 superagonist, N‐803) has also been shown to possess a longer half‐life and to more potently stimulate NK cells and T‐lymphocytes in vivo than unmodified IL15.^15^, ^16^ Clinical studies have demonstrated that N‐803 in combination with the anti‐PD‐1 monoclonal antibody nivolumab achieves good results in trial for patients with refractory metastatic non‐small cell lung cancer.^52^

The authors previously developed hIL15‐ABD, which is composed of human IL15 (hIL15) fused with an albumin‐binding domain (ABD)^53^ to preserve the immune‐stimulating effect of hIL15 and the high binding affinity to albumin to provide a prolonged half‐life in experimental mice.^14^ When combined with PD‐L1 antibodies, hIL15‐ABD demonstrates an enhanced anticancer effect on murine colon cancer and melanoma models. The tumour‐suppressing activity of hIL‐15‐ABD is correlated with the activation of NK and CD8^+^ T cells and a decrease in MDSCs and Tregs within the TME. Our in vivo results for hIL15‐ABD combined with Abraxane suggest that this immunotherapy might improve the anticancer effect of cytotoxic chemotherapy by activating the immune cells. The overexpression of NF‐κB may trigger immunosuppressive cascades to develop an immunosuppressive TME. In our IHC staining, the phosphorylation of NF‐κB was effectively suppressed by Abraxane combined with hIL15‐ABD.^54^, ^55^

hIL15‐ABD binds albumin with high affinity, and we hypothesized that it couples with Abraxane in nanoparticle form, which is composed of human albumin and paclitaxel at a ratio of 9:1.^56^ Our in vitro study showed that hIL15‐ABD couples to both human albumin and Abraxane with similar affinity (Figure 1A), indicating that this complex could form a bi‐functional nanotherapeutic that stimulates immunity and cytotoxicity. Paclitaxel induces immunogenic cell death (ICD) through increased trans‐localization of calreticulin (CRT)^57^ to the surface of the tumour cell and the release of high‐mobility group box 1 (HMGB1).^58^ This stimulates dendritic cells and increases the cytotoxicity of CD8^+^ T cells. Yang et al. demonstrated that paclitaxel regulates anticancer immunity by inducing ICD, and this effect is even more prominent in nano‐micelle‐encapsulated paclitaxel, possibly because there is improved tumour delivery and compatibility with the immune system.^40^ This study did not specifically determine the number of dendritic cells within the TME or TDLNs, but the IHC study showed that there was an increase in the number of one of the markers of antigen‐presenting cells, CD86, in both Panc02 and KPC tumours excised from mice treated with Abraxane, either alone or combined with hIL15‐ABD (Figure 4O).

The data show in vitro binding of hIL15‐ABD to Abraxane and enhanced anticancer immunity, both locally within the TME and systemically in the TDLN, spleen, and bone marrow. The superior anticancer effect of combinational therapy did not appear to increase the toxicity under our experimental protocols, as no discernible difference in haematoxylin and eosin‐stained normal organs, body weight and liver functioning among the various treatment groups. To better characterize the therapeutic window of hIL15‐ABD combined with Abraxane, animal single‐ or multiple‐dose‐escalating toxicity studies need to be carried out. Additionally, the extent to which the physical association of hIL15‐ABD and Abraxane contributes to the superior effect of the combination over each monotherapy remains unclear, although two experiments may clarify this issue: hIL15‐ABD and Abraxane could be intravenously injected into tumour‐bearing mice either separately or simultaneously to determine the anticancer efficacy and changes in the immune profiles within the TME and TDLN for these two groups of mice. It is also necessary to determine whether hIL15‐ABD bound to Abraxane exhibits better anticancer activity than N‐803 when these two modified IL15 therapeutics are individually combined with Abraxane. The results of this study show that Abraxane combined with hIL15‐ABD stimulates the anticancer activity of effector cells, inhibits immunosuppressive cells within the TME of murine PDAC, and provides a greater inhibitory effect than individual monotherapies.

## 5 CONCLUSION

Our study demonstrates a novel hIL15‐ABD that enhances the inhibitory effect of Abraxane on syngeneic murine PDAC subcutaneous Panc02 and orthotopic KPC models. hIL15‐ABD binds to Abraxane with high affinity to form a nanomedicine complex that has a cytotoxic and stimulating effect on PDAC and immune cells, respectively. The results of the flow cytometry demonstrate that there is enhanced accumulation and cytotoxicity for CD8^+^ T, NK cells and M1 macrophage and a decrease in the population of immunosuppressive Tregs and MDSCs. The data for the IHC‐stained TME are in agreement with that for the flow cytometry, in that there is enhanced anticancer immunity and decreased expression of immunosuppressive markers, such as Foxp3, IDO and VEGF. To the best of our knowledge, our data are the first to demonstrate that combining albumin‐binding therapeutics, such as hIL15‐ABD, could enhance the therapeutic effect of Abraxane. This finding could potentially lead to a strategy for generating a multi‐functional nanotherapy, with both tumour‐killing and immune‐stimulatory effects, using Abraxane as a delivery system. Although no discernible toxicity resulting from the addition of hIL15‐ABD was identified, further preclinical toxicity studies are essential to better characterize the therapeutic window of this novel combinational therapy.

## CONFLICT OF INTEREST

Keng‐Li Lan, Fei‐Ting Hsu and Chang‐Liang Tsai are co‐inventors of a patent application (WO2021031998A1) titled ‘Recombinant polypeptides and uses thereof’, which involves hIL15‐ABD.

## AUTHOR CONTRIBUTIONS

**Fei‐Ting Hsu:** Data curation (lead); Funding acquisition (lead); Methodology (lead); Project administration (equal); Supervision (equal); Validation (equal); Visualization (equal); Writing – original draft (equal); Writing – review & editing (equal). **Chang Liang Tsai:** Data curation (equal); Investigation (equal); Methodology (equal); Writing – original draft (equal). **I‐Tsang Chiang:** Data curation (equal); Methodology (equal); Writing – original draft (equal); Writing – review & editing (equal). **Keng‐Hsueh Lan:** Writing – original draft (equal). **Po‐Fu Yueh:** Data curation (equal); Investigation (equal). **Wen‐Yi Liang:** Data curation (supporting). **Chi‐Shuo Lin:** Data curation (equal). **Yee Chao:** Writing – original draft (equal). **Keng‐Li Lan:** Conceptualization (lead); Data curation (lead); Formal analysis (lead); Funding acquisition (lead); Investigation (equal); Methodology (equal); Project administration (equal); Resources (lead); Supervision (lead); Validation (equal); Visualization (equal); Writing – original draft (lead); Writing – review & editing (lead).

## Supporting information

Supplementary Material

## Data Availability

The original contributions presented in the study are included in the article/Supplementary Materials. Further inquiries can be directed to the corresponding author.
