# Supplementary material for: Synergistic effect of Abraxane that combines human IL15 fused with an albumin‐binding domain on murine models of pancreatic ductal adenocarcinoma
Source: J Cell Mol Med. 2022 Feb 17;26(7):1955–68. doi: 10.1111/jcmm.17220 (PMC8980892; doi:10.1111/jcmm.17220)
Supplement: Supplementary file 1 — Supplementary Material [file JCMM-26-1955-s001.docx]

The Synergistic Effect of Abraxane that combines human IL15 fused with an albumin-binding domain on murine models of pancreatic ductal adenocarcinoma

**Supplementary Materials**

**2.1. Antibodies and reagents**

Flow cytometry-related antibodies, such as CD4, CD8, CD11b, CD25, CD86, Foxp3, Gr-1, and IFN-γ, were all purchased from BD Pharmingen (San Diego, CA, USA). IHC-related antibodies, including CD8 (Sino Biological, Beijing, China), CD86 (Cell Signaling Technology, Danvers, MA, USA), NF-κB (ser536), Cleaved caspase-3, -8, and -9 (Cell Signaling Technology), Foxp3 (Elabscience), Granzyme B, IDO (Sigma-Aldrich, Louis, MO, USA) Ki-67, and VEGF (Elabscience), were purchased from the indicated companies. Abraxane was purchased from Taipei Veterans General Hospital Pharmacy. hIL15-ABD was expressed by transformed the BL21 (DE3) *E. coli* strain and purified as a hexa-histidine-tagged fusion protein from the inclusion body ^1^.

**2.2. Characterization for Abraxane binding of hIL15-ABD**

Human albumin and a molar equivalent of Abraxane (30 ng/well) were diluted in PBS and immobilized on a 96-well ELISA plate by incubation overnight at 4 °C. The albumin- and Abraxane-coated wells were blocked with 300 μL of 3% milk for 2 h at room temperature, followed by washing three times with 0.05% Tween-20 PBS. A succession of greater concentrations of hIL15-ABD were incubated with human albumin or Abraxane at room temperature for 1 h, followed by washing three times (0.05% Tween-20 PBS). The in vitro binding of his6-tagged hIL15-ABD to human albumin and Abraxane was detected using a HRP-tagged anti-his6 antibody and the addition of the HRP substrate, 3, 30, 5, 50-tetramethylbenzidine (100 μL/well). Peroxidase activity was inhibited after 20 min by adding 0.5 M H_2_SO_4_ (50 μL/well), and absorbance was measured at 450 nm using a microtiter plate reader.

**2.3. The stimulation of CTLL-2 cells by KPC-luc cells treated with Abraxane and hIL15-ABD**

The activation of the CTLL-2 T-lymphocyte cell line in the presence of KPC-luc cells treated with Abraxane and hIL15-ABD was determined by measuring the extent of CTLL-2 STAT-5 phosphorylation using flow cytometry. KPC-luc cells (5 × 10^3^ cells/well) were incubated with various concentrations of Abraxane (0, 100, 300, and 1000 nM) and hIL15-ABD (0, 100, 300, and 1000 nM ng/mL) for 4 h. When the medium was removed, CTLL-2 cells were added into KPC-luc on a 96-well plate (8 × 10^3^ cells/well), incubated for 30 min, and then fixed with formaldehyde (2% v/v final concentration) for 15 min at room temperature. The fixed cells were subjected to permeabilization, rehydration, and incubation with an antibody against phosphorylated STAT5 for 16 h at 4 °C, and then treated with FITC-conjugated anti-rabbit IgG for 60 min in the dark, using the technique from a previous study ^1^. Positive events were detected using a BD FACS Calibur flow cytometer and analyzed using CellQuest Pro and Cytexpert software (Beckman Coulter).

**2.4. Immune profiling of TILs, TDLN, and spleen and bone morrow with flow cytometry**

The immune profiles for CD4^+^T (Memory), CD8^+^ T, M1 macrophages, Tregs cells, and MDSCs in bone marrow (BM), the spleen ^2^, tumor-draining lymph nodes (TDLNs), and tumor-infiltrating lymphocytes (TILs) were determined using NovoExpress® flow cytometry (Agilent, Santa Clara, CA, USA). Cells isolated from mice tissue before CD8 and CD4 staining were first enriched by Mouse T Lymphocyte Enrichment Set-DM-BD Biosciences (BD Pharmingen). Memory T cells were characterized by CD4+ markers. The percentage and functions of activated CD8+ T cells were determined using CD8 and IFN-γ markers. Intracellular staining was conducted using a Fixation/Permeabilization Kit according to the manufacturer’s protocol ^3^. M1 macrophage cells from BM and TIL were characterized by CD11b^+^/CD86^+^ markers. The percentage of Tregs and MDSCs that was isolated from TDLN, SP, and BM was measured to determine the immunosuppressive function. Immunosuppressive cells were stained with anti-Foxp3-Alexa-Fluor-488 and CD4-PerCP-Cy™5.5/CD25-PE antibodies using a Mouse Treg Flow Kit according to manufacturer’s protocol. CD11b-FITC/Gr-1-PE antibodies were used to detect MDSCs. The percentage of each cell type was determined using FlowJo software ^1^.

**2.5. Animal experiments**

The animal experiments were approved by the Animal Care and Use Committee at China Medical University (approval number: CMU IACUC-2021-230) and Taipei Veterans General Hospital (approval number: TVGH IACUC-2021-052). Six-week-old female C57BL/6 mice were purchased from the National Laboratory Animal Center and housed in a pathogen-free animal facility. The xenograft model used mice that were inoculated in the right flank with Panc02 (2 × 10^5^ cells/mouse). Seven days after tumor inoculation, when the tumor had achieved a volume of 80–100 mm^3^, the mice were randomly divided into four groups (Figures 2A and 3A): A vehicle control (0.1% DMSO, *n* = 5), Abraxane (200 μg, *n* = 4), hIL15-ABD (5 μg, *n* = 4), and a combination of Abraxane and hIL15-ABD (*n* = 6). All mice received intravenous injections twice a week for two weeks. The tumor volume was measured using a caliper and was calculated as: Volume = Length × Width^2^ × 0.523.

For the orthotopic pancreatic tumor model, KPC cells were trypsinized, resuspended in PBS, and mixed with Matrigel at a ratio of 1:1 to a final concentration of 1 × 10^4^ cells/μL. Each C57BL/6J mouse was anesthetized with 1%–2% vaporized isofluorane, which was delivered through a nosecone during surgery. The pancreatic tail of each mouse was exposed using a 0.5–1 cm cut and injected with 5 × 10^5^ KPC cells in 50 μL of a 1:1 PBS/Matrigel mixture. The pancreas of the mouse was gently pushed back into the peritoneal cavity, and the abdominal wall was closed using a 4-0 vicryl suture. The mice were divided randomly into four groups (*n* = 3 each), and treatment was initiated eight days after tumor inoculation by intravenous injections of the control, Abraxane, hIL15-ABD, and the combination (Figure 3A).

**2.6. PET/MRI imaging**

On the 19th day after orthotopic inoculation of KPC cells, the mice were anesthetized and placed in a birdcage MRI coil. The MRI sequences included 0.5 mm-thick Axial T2 Turbo RARE images (TR = 5920 ms, TE = 36 ms, resolution = 0.078 × 0.078f, average = 6, slice number = 50). For PET imaging, the mice were injected in their tail vein with [^18^F]FDG (7.03–8.1 MBq), followed by 1 h of FDG uptake, before scanning. After manual segmentation, static PET images were acquired using a 7T PET/MR Inline (Bruker, Rheinstetten, Germany) for 10 min with an energy window of 357–664 keV. LIFEx^4^ was used to calculate the mean SUV for gross pancreatic tumors.

**2.7. Blood biochemical tests**

Blood samples of approximately 0.6 mL/kg were withdrawn from the heart on Day 25 for the Panc02 subcutaneously inoculated model. Blood samples were stored in vacutainers without anticoagulant for biochemical analysis of aspartate aminotransferase (AST/GOT) and alanine aminotransferase (ALT/GPT). Sera were separated by centrifugation at 800× *g* for 10 min and stored at –80 °C, prior to analysis ^5^.

**2.8. Hematoxylin and eosin (H&E) staining and Immunohistochemistry (IHC)**

Samples from the heart, lungs, liver, kidneys, intestines, pancreas, and tumor were fixed using formalin, embedded using paraffin, and sliced using a paraffin microtome. Slices were subjected to hematoxylin and eosin (H&E) and immunohistochemistry (IHC) staining. The H&E and IHC staining followed the protocols described in a previous study ^6^. Stained slices were acquired using a Nikon ECLIPSE Ti-U microscope at a 200× magnification. The quantification results for the protein expression on the IHC slides were analyzed using the ImageJ software (National Institutes of Health, Bethesda, MD, USA).

**References**

1. Hsu FT, Liu YC, Tsai CL, et al. Preclinical Evaluation of Recombinant Human IL15 Protein Fused with Albumin Binding Domain on Anti-PD-L1 Immunotherapy Efficiency and Anti-Tumor Immunity in Colon Cancer and Melanoma. *Cancers (Basel)*. 2021; 13.

2. Moschetta M, Pretto F, Berndt A, et al. Paclitaxel enhances therapeutic efficacy of the F8-IL2 immunocytokine to EDA-fibronectin-positive metastatic human melanoma xenografts. *Cancer Res*. 2012; 72: 1814-24.

3. Hsu FT, Chen TC, Chuang HY, et al. Enhancement of adoptive T cell transfer with single low dose pretreatment of doxorubicin or paclitaxel in mice. *Oncotarget*. 2015; 6: 44134-50.

4. Nioche C, Orlhac F, Boughdad S, et al. LIFEx: A Freeware for Radiomic Feature Calculation in Multimodality Imaging to Accelerate Advances in the Characterization of Tumor Heterogeneity. *Cancer Res*. 2018; 78: 4786-9.

5. Parasuraman S, Raveendran R, Kesavan R. Blood sample collection in small laboratory animals. *Journal of pharmacology & pharmacotherapeutics*. 2010; 1: 87-93.

6. Weng MC, Li MH, Chung JG, et al. Apoptosis induction and AKT/NF-κB inactivation are associated with regroafenib-inhibited tumor progression in non-small cell lung cancer in vitro and in vivo. *Biomed Pharmacother*. 2019; 116: 109032.
